# Recent advances of exosomes in age-related macular degeneration

**DOI:** 10.3389/fphar.2023.1204351

**Published:** 2023-06-02

**Authors:** Feng Gu, Jiyu Jiang, Peng Sun

**Affiliations:** Department of Ophthalmology, The First Hospital of China Medical University, Shenyang, China

**Keywords:** age-related macular degeneration, exosomes, pathogenesis, diagnosis, treatment

## Abstract

Exosomes are 30–150 nm extracellular vesicles that are secreted by almost all types of cells. Exosomes contain a variety of biologically active substances, such as proteins, nucleic acids, and lipids, and are important in the intercellular communication of biological mediators involved in nerve injury and repair, vascular regeneration, immune response, fibrosis formation, and many other pathophysiological processes. Although it has been extensively studied in the field of cancer, the exploration of ocular diseases has only just begun. Here, we discuss the latest developments in exosomes for age-related macular degeneration (AMD), including the pathogenesis of exosomes in age-related macular degeneration, their potential as diagnostic markers, and therapeutic vectors of the disease. Finally, the study of exosomes in age-related macular degeneration is still relatively few, and more detailed basic research and clinical trials are needed to verify its application in treatment and diagnosis, so as to adopt more personalized diagnosis and treatment strategies to stop the progression of age-related macular degeneration.

## 1 Introduction

Age-related macular degeneration (AMD) is a degenerative eye disease, which is mainly divided into dry and wet types, and is one of the main causes of blindness among the elderly throughout the world (Fleckenstein et al., 2021; Murray et al., 2022). AMD not only causes great suffering to patients and their families but also seriously affects the quality of human life (Armento et al., 2021; Sarkar et al., 2022). To date, the main treatment is ocular injection of anti-VEGF drugs, but this needs to be repeated several times and the results are not satisfactory (Solomon et al., 2019; Parravano et al., 2021).

Exosomes have the characteristics of double-layer membrane structure, long circulation time and low biodegradability, which can protect the contents from extracellular degradation. (Kalluri and Lebleu, 2020; Yang et al., 2020; Lai et al., 2022). Additionally, because of their nanometer size and the low content of membrane-bound proteins, the immunogenicity of exosomes is low. Therefore, exosomes can deliver their inclusions to target cells through pinocytosis, phagocytosis and membrane fusion to facilitate intercellular communication (Yang et al., 2019; Noonin and Thongboonkerd, 2021) The contents of exosomes can be delivered to themselves or nearby cells, as well as to distant tissue cells through blood flow (Pegtel and Gould, 2019; Han et al., 2022), so they have been widely studied in the field of cancer (Sun et al., 2018a; Meng et al., 2019). However, exploration of exosomes in ocular diseases has only begun. In this review, we highlight and discuss the latest research advances on exosomes in AMD, including their pathogenic mechanisms and their potential as diagnostic markers and therapeutic vectors. However, there is still no complete and standardized system for the isolation and extraction of exosomes, so there is an urgent need for a complete strategy to obtain large amounts of highly purified exosomes with stable functions, in order to adopt more diagnosis strategies and personalized treatment to stop disease progression.

## 2 Overview of AMD

Vision loss and blindness are health problems that the elderly cannot ignore. AMD is a chronic degenerative retinal disease characterized by progressive and irreversible loss of central vision, which is the leading cause of blindness in people aged 50 years and older (Kaarniranta et al., 2020; Stahl, 2020; Fleckenstein et al., 2021). With increasing life expectancy, AMD has become a major public health concern. AMD begins with the formation of lipoprotein-rich deposits in the macula, and has two main clinical manifestations: atrophic (dry) AMD and neovascular (wet) AMD (Mitchell et al., 2018; Chakravarthy and Peto, 2020; Fleckenstein et al., 2021). Dry AMD is non-exudative and is characterized by focal atrophy of retinal pigment epithelium (RPE) cells and loss of macular photoreceptors (Handa et al., 2019; Gupta et al., 2023). Wet AMD is exudative and is characterized mainly by the formation of choroidal neovascularization (Ricci et al., 2020). Patients with dry AMD experience a slight loss of vision with distortions or blind spots in or around the central vision. However, patients with wet AMD may experience severe vision loss due to choroidal neovascularization involved (Sarkar et al., 2022).

The development of AMD is multifactorial and is mainly associated with age and environmental and genetic factors. Among genetic factors complement is considered to be a key component of the pathogenesis of AMD. The association between single nucleotide polymorphisms (SNPs) in the complement factor H and increased risk of AMD has been confirmed (Toomey et al., 2018). Variation in complement factor C3 also contributes to the pathogenesis of AMD (Park et al., 2019). In addition, smoking, alcohol consumption, and a high-cholesterol diet can also increase the risk of the disease. Together, these factors initiate and accelerate AMD development (Kauppinen, 2020). In the early stages of AMD, abnormal mitochondrial function occurs due to the presence of hyperoxia tension and chronic light exposure, which induces damage to RPE cells located in the outer layer of the retinal barrier, leading to a decrease in cellular autophagy, which in turn activates the immune and inflammatory responses (Behnke et al., 2020; Armento et al., 2021). Large amounts of inflammatory and vascular endothelial growth factors lead to RPE cell apoptosis and abnormal angiogenesis, which ultimately lead to vision loss (Kaarniranta et al., 2023). In summary, the inability of RPE cells to adapt to the aging process or stressful conditions leads to AMD development.

Currently, anti-vascular endothelial growth factors are used mainly for the treatment of wet AMD (Voelker, 2021; Sarkar et al., 2022). However, long-term use of anti-vascular endothelial growth factor not only decreases efficacy but may also lead to retinal hemorrhage (Fabre et al., 2022). Up to now there is no specific treatment for dry AMD (Stahl, 2020). Therefore, studies addressing the mechanisms by which AMD occurs are of great interest for the adoption of more personalized treatment and diagnostic strategies to stop disease progression.

## 3 Occurrence and mode of action of exosomes

**FIGURE 1:**
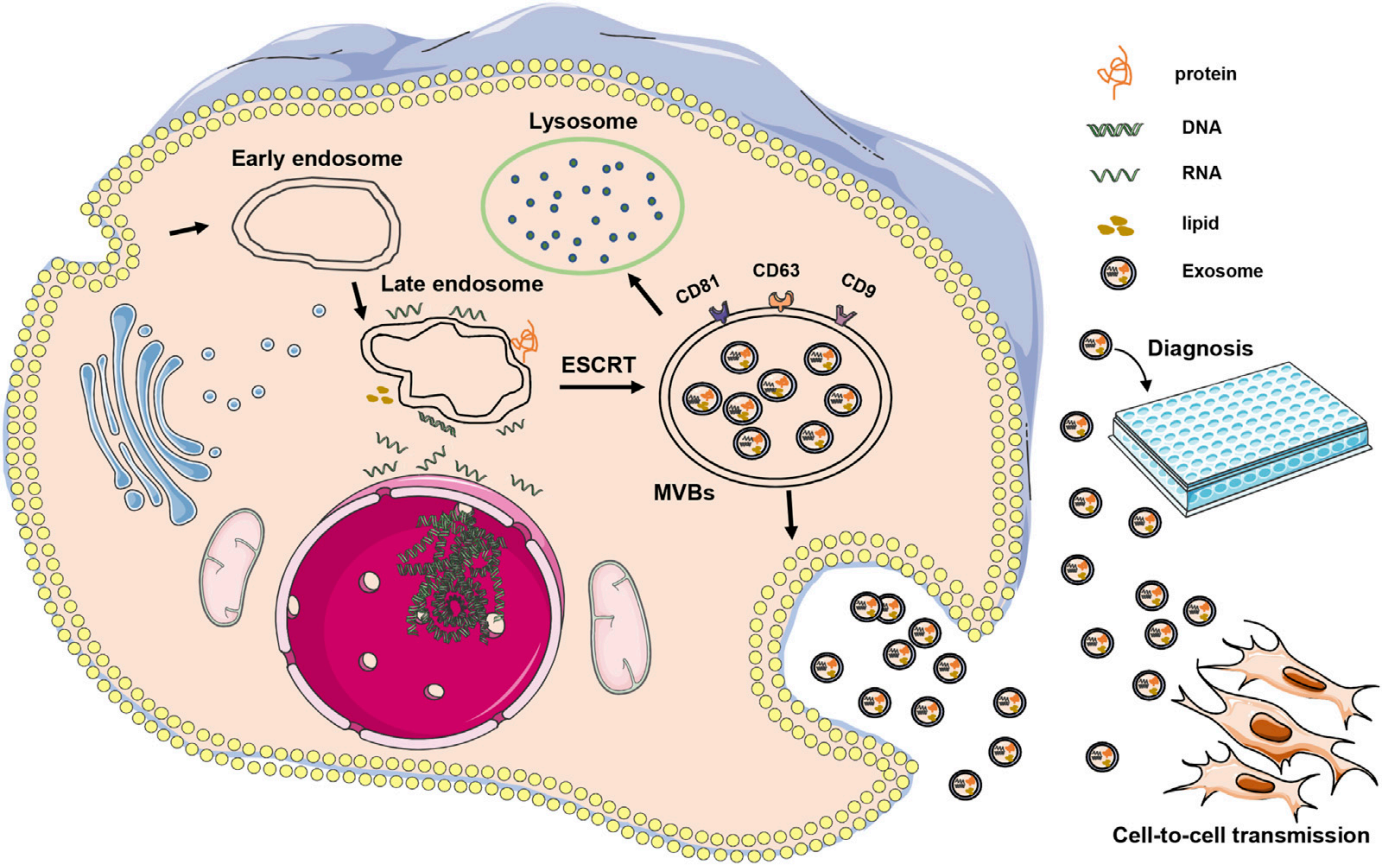
Illustrates the occurrence and mode of action of exosomes. Exosomes are initially formed through inward budding from cell membranes, resulting in the development of early endosomes, which then mature into late endosomes. The endosomal sorting complex required for transport mechanism facilitates the gradual accumulation of intraluminal vesicles in late endosomes, leading to the formation of MVBs. These MVBs can either fuse with lysosomes or release their contents outside the cell to form stable exosomes. In AMD, exosomes can be transmitted between cells, potentially contributing to the onset or treatment of diseases. Additionally, exosomes can serve as a diagnostic tool.

The exosome were first discovered in sheep erythrocytes cultured *in vitro* as microvesicles with size of 30–150 nm (Ahn and Johnstone, 1993; Yang et al., 2020). The exosomes first bud inward from the cell membranes containing ubiquitinated surface receptors, leading to the formation of early endosomes. With the help of the Golgi apparatus, these early endosomes mature into late endosomes. The classical mechanism of the endosomal sorting complex required for transport allows gradual accumulation of intraluminal vesicles in late endosomes, leading to the formation of multivesicular bodies (MVBs) (Kalluri and Lebleu, 2020; Yang et al., 2020). MVBs eventually have two pathways: one fusing with lysosomes, leading to the breakdown of their contents, and the other fusing with the plasma membrane and releasing their contents outside the cell to form stable exosomes with double-layer membrane structure (Song et al., 2019; Han et al., 2022) (Figure 1). Therefore, exosomes contain proteins, lipids, and nucleic acids from their parent cells, and exosomes of different cellular origins have different functions (Pegtel and Gould, 2019; Yu et al., 2021). They are secreted from cells, released into body fluids (e.g., blood, tears, aqueous fluid, vitreous humor, urine, and spinal fluid), and transported between cells via the circulatory system (Sun et al., 2018b; Crescitelli et al., 2021).

Exosomes can facilitate intercellular communication by delivering messages to target cells via pinocytosis, phagocytosis, and membrane fusion (Wortzel et al., 2019; Kalluri and Lebleu, 2020). They have been extensively studied in various biological processes such as cell proliferation, apoptosis, autophagy, inflammation, and angiogenesis (Bebelman et al., 2018; Couch et al., 2021; Kimiz-Gebologlu and Oncel, 2022). Cardiac telocytes inhibit cardiac microvascular endothelial cell apoptosis through exosomal miRNA-21-5p-targeted cdip1 silencing to improve angiogenesis following myocardial infarction (Liao et al., 2021). Umbilical mesenchymal stem cell-derived exosome-encapsulated hydrogels accelerate bone repair by enhancing angiogenesis (Zhang et al., 2021). Matrix metalloproteinases, membrane-linked proteins, and proteoglycans in exosomes can also remodel the extracellular matrix to increase tumor metastasis (Genschmer et al., 2019; Mohan et al., 2020). Since angiogenesis and extracellular matrix remodeling are also major pathogenic mechanisms in AMD, exosomes may also play a very important pathogenic role in AMD. At the same time, the inclusions of exosomes respond to the status of their parent cells. At present, exosomes have been widely used as biomarkers in the field of cancer. For example, Techniques have been developed to analyze proteins and nucleic acids from serum exosomes for early cancer detection and surveillance in cancer screening (Zhu et al., 2020; Tang et al., 2021), so exosomes may have a place in the diagnosis of AMD. Finally, the stable and packable biological properties of exosomes make them essential in AMD therapeutic studies. Because of these functions and properties, the role of exosomes in AMD should be studied in more depth.

## 4 Pathogenic role of exosomes in AMD

The early onset of AMD is due to the accumulation of lipids and proteins, resulting in deposits under the RPE cells, which are located between the bloodstream of the choroid and the outer segments of the photoreceptors and secrete a large number of factors to maintain a balance between these tissues (Brown et al., 2019; Kaarniranta et al., 2020). Since exosomal inclusions reflect the state of their parent cells, any change in the parent cells may alter the molecular composition of exosomes (Ke et al., 2020; Zhang et al., 2023). Exosomal inclusions of RPE cells during AMD may reflect the pathophysiological state of RPE cells (Ke et al., 2020; Zhang et al., 2023). It has been reported that RPE cells can increase the release of exosomes during stressful states, and the secreted exosomes contain macular-related proteins and proteins involved in the immune response (Atienzar-Aroca et al., 2016; Knickelbein et al., 2016; Ke et al., 2020; Flores-Bellver et al., 2021; Du H et al., 2022), and miRNA (Morris et al., 2020; Du SW and Palczewski, 2022), which are also detected in AMD patients (Flores-Bellver et al., 2021), suggesting that the formation of sub-RPE cell deposits may be derived from RPE cell exosomes. In addition, RPE under stress during AMD releases large amounts of VEGFR2-containing exosomes, which increase the tube-forming capacity of endothelial cells, promote neointima formation, and facilitate the development and progression of wet AMD (Sahaboglu et al., 2019).

It has been suggested that increased exosome release by RPE cells in response to external stress may initially be a protective mechanism, since anti-angiogenic, anti-inflammatory, and antioxidant components, as well as proteins that play a neuroprotective role in RPE and photoreceptor cells, have been identified in exosomes (Brown, 1994; Kelly et al., 1994). The overproduction and secretion of these proteins leads to continuous proliferation of toxic forms, such as alpha-synuclein and nerve poison (Alvarez-Erviti et al., 2011; Sardar et al., 2018), leading to RPE cell dysfunction and the formation of sub-RPE cell deposits (Bjordal et al., 2020).

In RPE cells, exosomes are released from both the apical and basolateral sides, and it has been reported that under stress conditions in RPE cells during AMD, most RPE cells secrete exosome proteins that differ significantly between the apical and basolateral sides (Klingeborn et al., 2017; Klingeborn et al., 2018; Flores-Bellver et al., 2021), indicating the specificity of exosome release from both ends of polarized cells, as neither apical nor basolateral detection alone can accurately account for RPE cell-secreted exosome components. Therefore, it is important to clarify exactly which side of the RPE cell secretes the exosomes to guide the study of the AMD disease process. Elucidating the pathogenic mechanisms of exosomes in the AMD disease process and determining the exact factors in exosomes in AMD are essential for the development of more personalized therapeutic and diagnostic strategies.

## 5 Diagnostic role of exosomes in AMD

Exosomes widespread presence in body fluids and have relatively noninvasive collection methods. The lipid bilayer outermost also has a good protective effect on nucleic acids, lipids and protein contained therein. And these inclusions in exosomes can change according to the metabolic conditions of the body, so exosomes can also serve as ideal biomarkers for the detection, monitoring and predicting of diseases (Liu et al., 2018; Yu et al., 2021; Yu et al., 2022). Exosomes are abundant in tear fluid (Han et al., 2021; Huang et al., 2023), aqueous fluid (Lande et al., 2020; Zhang et al., 2022), vitreous fluid (Tsai et al., 2021; You et al., 2023), and blood (Li et al., 2017; Wortzel et al., 2019). These liquids are all important body fluids associated with eyes. Although not much has been reported, identification of exosome-specific biomarkers for ocular diseases is of great importance. Thus, the detection of nucleic acids and proteins from isolated circulating exosomes provides real-time insights into the development of AMD, which is not possible with other methods such as fundus imaging or optical coherence tomography imaging.

Among these important body fluids related to eye diseases, aqueous humor contains abundant exosomes, and the exosomes in aqueous humor contain many bioactive substances secreted from other intraocular cells. Studies have examined the exosome proteins in aqueous humor from AMD. Comparing the protein profiles of AMD and the control group, six types of proteins showed obvious enrichment in AMD patients. Three of them, Apolipoprotein A-I, Clusterin and Complement C3, were thought to be related to AMD in previous studies. This suggests that exosome proteins extracted from the aqueous fluid of patients with AMD may serve as new diagnostic biomarkers for AMD (Kang et al., 2014; Dismuke et al., 2015). Although vitreous humor does not contain any cell structure, the exosomes at the back of it come mainly from retinal epithelial cells and vascular endothelial cells, indicating that there is a certain degree of communication between these eye tissues (Zhao et al., 2018). Pigment epithelium-derived factor in vitreous humor has been proved to be a biomarker of neovascular AMD (Yokoi et al., 2007). Therefore, aqueous humor and vitreous humor can specifically reflect the characteristics of eye disease, and can be used as potential biomarkers of AMD. However, the process of obtaining aqueous humor and vitreous humor is relatively complicated and needs to be obtained by needle biopsy or surgery.

Besides, AMD-associated exosomes are not only present in eye-associated body fluids but are also present in blood. And the method of obtaining blood is relatively non-invasive and abundant. Because RPE cells are highly polarized epithelial cells, they constitute the retinal barrier of the eye. Polarization leads to the targeted secretion of exosomes. Exosomes released from the base of RPE cells enter the systemic circulation through the choroid, and the released exosomes can also be separated from the blood. To test this point, researchers isolated exosomes from the basal part of RPE cells and determined their protein group content. It is confirmed that the exosomes released by RPE cells can be separated from blood (Locke et al., 2014; Klingeborn et al., 2018). Therefore, RPE cell-derived exosomes in the blood may provide a diagnostic indicator of AMD (Klingeborn et al., 2017). RPE cell-derived exosomes have been reported to secrete autophagy-associated proteins such as calpain-2 *in vivo*, which may also serve as a possible diagnostic indicator of AMD (Zhang et al., 2023).

MiRNAs in exosomes can also be used as potential biomarkers of AMD. Some miRNAs are associated with angiogenesis, inflammation, and oxidative stress, suggesting that they may be potential target for AMD (Zhao et al., 2020; Liu et al., 2022). MiR-486–5p, miR-124, miR-21, miR-146b, Let-7 clusters, and miR-885–5p, differ significantly in controls and individuals with AMD (Chu-Tan et al., 2018; Wooff et al., 2020). The results showed that miR-486–5p, miR-124 and Let-7 clusters were increased in serum samples from AMD patients, whereas the expression of miR-21, miR-146b, and miR-885–5p was downregulated. The miR15/107 cluster, miR17∼92 cluster, miR-21, miR-132, miR-296, miR378, and miR-519c clusters have been reported to play an important role in angiogenesis (Cha et al., 2010; Turco et al., 2020; Chiba et al., 2021; Ghaffari-Makhmalbaf et al., 2021) and could serve as potential biomarkers for AMD.

Exosome biomarkers can be used not only to assess disease severity but also to monitor disease progression. Anti-VEGF therapy is generally used in clinical practice to control the progression of wet AMD (Mettu et al., 2021). When comparing before and after continuous anti-VEGF injections of ranibizumab, two exosome proteins, SERPINA1 and AZGP1, were found to decrease with time after treatment (Tsai et al., 2022). Thus, VEGF induces these proteins to promote the development of AMD. Although the expression of these two proteins may respond to AMD treatment by detecting them, more basic studies and clinical data are still needed to demonstrate the role of exosome markers in AMD surveillance.

In general, with the continued progress and refinement of exosome-specific isolation techniques and their identification methods, exosomes will become ideal and less invasive biomarkers for the detection, monitoring, and prediction of AMD. Therefore, it is important to study exosomes as biomarkers of AMD. The diagnostic role of exosomes in AMD is summarized in Table 1.

**TABLE 1:** A brief description of the expression level of exosomes in AMD.

Specific disease of the patient	Exosomes source	Exosomal content	Expression level	Ref.
Neovascular AMD	Vitreous humor	Pigment epithelium-derived factor	Increase	Yokoi et al. (2007)
Neovascular AMD	Aqueous humor	APOA1, CLU, C3	Increase	Kang et al. (2014)
Atrophic AMD	Retinal pigmented epithelium cell	αB-crystallin	Increase	Locke et al. (2014), Klingeborn et al. (2018)
Atrophic AMD	Retinal pigmented epithelium cell	calpain-2	Increase	Ran et al. (2020), Zhang et al. (2023)
Neovascular AMD	Mice retinal	miR-21,miR-146b, miR-885–5p	Decrease	Wooff et al. (2020)
Neovascular AMD	Mice retinal	miR-486–5p, miR-124 and Let-7 clusters	Increase	Wooff et al. (2020)
Neovascular AMD	Vitreous humor	SERPINA1,AZGP1	Increase	Dismuke et al. (2015)

## 6 Therapeutic role of exosomes in AMD

Current therapies for AMD, such as photodynamic therapy, vitreous surgery, radiation therapy, and medication, are not very effective (Ammar et al., 2020; Khanani et al., 2022). Anti-vascular endothelial growth factor is currently the newest and most effective treatment for wet AMD and has improved the vision of patients with AMD to some extent (Parravano et al., 2021). However, anti-vascular endothelial growth factor therapy is ineffective in some patients with advanced wet AMD. Furthermore, some patients develop more severe visual impairment after anti-vascular endothelial growth factor treatment (Tranos et al., 2013; Yang et al., 2016; Mettu et al., 2021). Therefore, more effective alternative therapies are needed. Delivering therapeutic agents to the posterior part of the eye is difficult because of the specific anatomy and physiology of the eye and the presence of various barriers. Exosomes have advantages over other carriers because of their nanoscale size, low immunogenicity, long circulation time, and low biodegradability, both to promote cellular uptake and to protect inclusions from extracellular degradation (Kalluri and Lebleu, 2020; Yang et al., 2020; Zhang et al., 2020). Most importantly, exosomes can easily cross the retinal barrier and target damaged cells (Harrell et al., 2022; Zhang et al., 2022), making them an ideal tool for AMD treatment.

As stem cells play an important role in tissue repair, most studies on the treatment of AMD by exosomes is based on stem cell exosomes. For example, miR-27b in human umbilical cord MSC-derived exosomes reduces retinal fibrosis by targeting HOXC6 to inhibit the epithelial-mesenchymal transition (Li et al., 2021). Human umbilical cord MSC-derived exosomes can improve the function of RPE cells under blue light irradiation and reduce laser retinal damage by downregulating VEGF-A (He et al., 2018). Bone marrow MSC-derived exosomes can also exhibit anti-inflammatory effects by delivering miRNA-126 (Zhang et al., 2019). In addition, studies have also reported that intravitreal injection of MSC-derived exosomes could improve retinal laser injury by reducing damage and inhibiting apoptosis and inflammation (Yu et al., 2016; Ma et al., 2020).

Neovascularization is the main cause of wet AMD. Many research groups have demonstrated the antiangiogenic properties of exosomes in AMD. For example, periocular injection of retinal astrocyte exosomes reduced CCL2-dependent migration of macrophages to the lesion site and attenuated angiogenesis (Hajrasouliha et al., 2013). As CCL2 is considered a key chemokine in the pathogenesis of several retinal degenerative diseases (Zhu et al., 2017; Badia et al., 2021) and is a known target of miR-124–3p (Zuzic et al., 2019), exosome-based supplementation with miR-124–3p may be an effective therapy. In addition, the injection of retinal astrocyte-derived exosomes into the vitreous of mice with retinopathy showed significant protective effects, reducing avascular areas, VEGF expression, and photoreceptor cell apoptosis in the retina (Zhao et al., 2018). Thus, neovascularization in wet AMD can be reduced by adding therapeutic exosomes. These studies suggest that exosomes can be used for the treatment of wet AMD.

Finally, exosomes containing therapeutic agents have been shown to have better efficacy and bioavailability (Munagala et al., 2016; Kojima et al., 2018). Therefore, researchers are attempting to load various drugs into exosomes to take advantage of them for drug delivery to the target site. Exosomes loaded with drugs show higher efficacy than free drugs (Gao et al., 2018; Ran et al., 2020), which provides a new idea for AMD drug therapy. We speculate that future studies will apply exosomes with anti-angiogenic, anti-inflammatory, neuroprotective, and proliferative effects and combined with drug-targeted delivery approaches, such engineered exosomes may become a viable therapy for AMD. Therefore, more in-depth studies are needed to load exogenous molecules and drugs into exosomes to target AMD and provide personalized and efficient therapies. The therapeutic role of exosomes in AMD is summarized in Table 2.

**TABLE 2:** A brief description of the therapeutic role of exosomes in AMD.

Cell line/tissue	Exosomes source	Exosomal content	Therapeutic role	Ref.
Human umbilical cord bone marrow cell	MSC	miR-27b	Reduces retinal fibrosis	Li et al. (2021)
Human umbilical cord bone marrow cell	MSC	Not sure	downregulating VEGF-A	He et al. (2018)
Human umbilical cord bone marrow cell	MSC	miRNA-126	anti-inflammatory	Zhang et al. (2019)
Human umbilical cord bone marrow cell	MSC	Not sure	Inhibiting MCP-1	Yu et al. (2016), Ma et al. (2020)
Rat bone marrow cell	MSC	Not sure	reducing damage and inhibiting apoptosis and inflammation	Ma et al. (2020)
Human umbilical cord cell/Mouse adipose tissue	MSC	MCP-1	reducing damage and inhibiting apoptosis and inflammation	Yu et al. (2016)
Retinal astrocyte cell	cell	CCL2	inhibiting angiogenesis	Hajrasouliha et al. (2013)
Not sure	Not sure	miR-124–3p	inhibiting angiogenesis	Zuzic et al. (2019)

## 7 Conclusion and perspective

In summary, exosomes contain a variety of proteolipids and nucleic acids that contribute to functions such as information transfer, immune regulation, repair, and regeneration. The etiology of AMD involves immune cell activation, inflammation, neuronal degeneration, neovascularization, and fibrosis; therefore exosomes play an important role in the disease process of AMD. In addition, exosomes can cross biological barriers and be used as therapeutic carriers or biomarkers.

**FIGURE 2:**
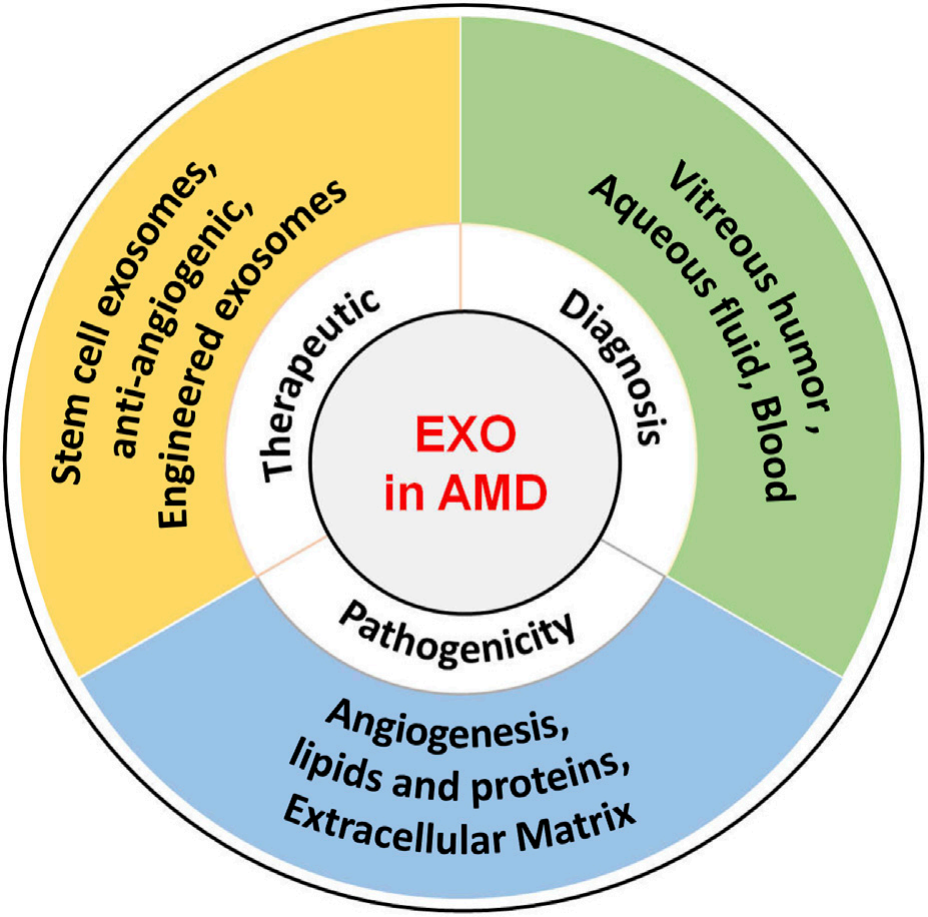
Role of exosomes in AMD. The role of exosomes AMD encompasses their potential to induce pathogenesis, serve as diagnostic biomarkers, and function as therapeutic carriers.

Interesting, exosomes are a double-edged sword in AMD, as they can promote disease progression and play a therapeutic role. For example, exosomes from RPE cells under stress can lead to disease progression, whereas exosomes from retinal astrocytes can inhibit angiogenesis, a treatment for AMD. These two play opposing roles. However, exosomes remain relatively poorly studied in AMD, and more detailed basic research and clinical trials are required to validate their therapeutic and diagnostic applications. Furthermore, in order to translate successfully into clinical treatment, there is an urgent need for advanced technologies to obtain large amounts of highly purified and functionally stable exosomes to provide patients with personalized therapeutic and diagnostic programs. The role of exosomes in AMD is summarized in Figure 2.
